# A Short History of Three Cases of Women, Who Were Inoculated for the Small-pox during Pregnancy

**Published:** 1784

**Authors:** 


V. *A ſhort Hiſtory of three Caſes of Women, who were inoculated for the Small-pox during Pregnancy. By Mr.* Benjamin Roberts, *Surgeon at*

*at Salisbury. Communicated to Dr.* Simmons *by Dr.* Denman.

MARY Hickes, near the ninth month of her pregnancy, was inoculated on the 14th day of November, 1783. Her arm inflamed, and every appearance promised the most favourable termination to the disease. On the 21st, the eruptive fever commenced, and the small-pox, which were not very numerous, came out on the third day. When these began to dry, she had one violent rigor, followed by other symptoms, which denoted the death of the child. Being costive, an opening medicine was given, by which, and some diaphoretics, she was much relieved. On the 28th, she fell into labour, and on the 29th, was delivered of a dead child. The body of the child was covered with the small-pox; the bases of which were in a gangrenous state.

Elizabeth Boon, in the eighth month of her pregnancy, was inoculated on the 15th of November, 1784. On the 20th, the eruption appeared, and the fever did not abate. Six ounces of blood were taken away, and a saline mixture was given. On the 27th, her pains came came on, and ſhe was in a few hours delivered of a living child. There was not the leaſt trace of eruptions on any part of the body of the child, which died in about ten days with a complaint in its bowels.

Mary Jeffery, in the eighth month of her pregnancy, was inoculated on the ſame day with Elizabeth Boon. She paſſed through all the ſtages of the diſeaſe with as little diſturbance as any perſon I ever ſaw; and three weeks afterwards was delivered of a living child, without any appearance of the diſeaſe upon it.

I was induced to inoculate theſe patients by their own earneſt deſire, and becauſe it was ſcarcely poſſible for them to eſcape the natural infection, the diſeaſe being at that time ſpread through almoſt every family in Wilton, the place where they lived.

